# Role of Nicotinamide in Genomic Stability and Skin Cancer Chemoprevention

**DOI:** 10.3390/ijms20235946

**Published:** 2019-11-26

**Authors:** Luca Fania, Cinzia Mazzanti, Elena Campione, Eleonora Candi, Damiano Abeni, Elena Dellambra

**Affiliations:** 1 First Dermatology Clinic, IDI-IRCCS, via dei Monti di Creta 104, 00167 Rome, Italy; l.fania@idi.it (L.F.); c.mazzanti@idi.it (C.M.); 2 Dermatologic Unit, University of Rome Tor Vergata, Viale Oxford 81, 00133 Rome, Italy; campioneelena@hotmail.com; 3 Department of Experimental Medicine, University of Rome Tor Vergata, Via Montpellier, 1, 00133 Rome, Italy; candi@uniroma2.it; 4 Biochemistry Laboratory, IDI-IRCCS, 00167 Rome, Italy; 5 Clinical Epidemiology Unit, IDI-IRCCS, via dei Monti di Creta 104, 00167 Rome, Italy; d.abeni@idi.it; 6 Laboratory of Molecular and Cell Biology, IDI-IRCCS, via dei Monti di Creta 104, 00167 Rome, Italy

**Keywords:** keratinocytes, non-melanoma skin cancers, skin aging, nicotinamide

## Abstract

Nicotinamide (NAM) is an amide form of vitamin B3 and the precursor of nicotinamide adenine dinucleotide (NAD+), an essential co-enzyme of redox reactions for adenosine triphosphate (ATP) production and for other metabolic processes. As NAD+ status is critical in maintaining cellular energy, vitamin B3 deficiency mainly affects tissues that need high cellular energy causing pellagra and skin sun sensitivity. In animal models, NAD+ deficiency leads to UV sensitivity of the skin, impairs DNA damage response, and increases genomic instability and cancer incidence. Furthermore, NAD+ depletion is associated with human skin aging and cancer. NAM prevents the UV-induced ATP depletion boosting cellular energy and enhances DNA repair activity in vitro and in vivo. Moreover, NAM reduces skin cancer incidence and prevents the immune-suppressive effects of UV in mice. Thus, NAM is involved in the maintenance of genomic stability and may have beneficial effects against skin aging changes and tumor development. Clinical studies showed that topical use of NAM reduces cutaneous aging. Furthermore, oral NAM administration reduces the level of UV-mediated immunosuppression and lowers the rate of non-melanoma skin cancers in high-risk patients. Therefore, NAM replenishment strategy may be a promising approach for skin cancer chemoprevention.

## 1. Introduction

**Figure 1:**
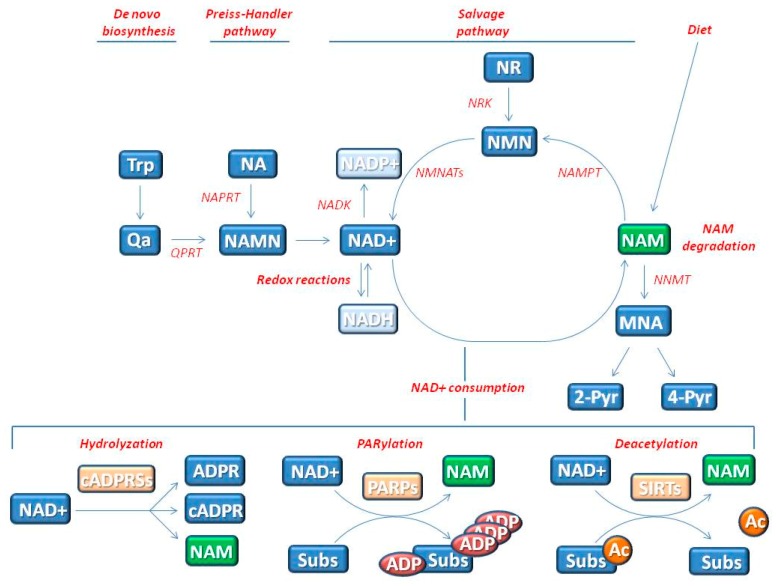
Nicotinamide (NAM) and Nicotinamide Adenine Dinucleotide (NAD+) metabolism. One source of NAM is the diet, via intake of eggs, meat, fish, and mushrooms. After ingestion, NAM is readily absorbed from the gastro-intestinal tract and widely distributed in the body tissues. NAM is the precursor of nicotinamide adenine dinucleotide (NAD+) that is an essential co-enzyme of redox reactions for adenosine triphosphate (ATP) production and for several metabolic processes. NAM is converted to NAD+ through the salvage pathway that represents the major route of NAD+ biosynthesis in mammals. NAM phosphoribosyltransferase (NAMPT) is the rate-limiting enzyme that catalyzes the first step in the biosynthesis of NAM mononucleotide (NMN) from NAM. Subsequently, the NMN adenylyltransferases (NMNATs) utilize ATP for the generation of NAD+. This coenzyme may be directly converted from NADP+ by NAD kinase (NADK). NMN can be also synthetized by nicotinic acid (NA) and NAM riboside (NR). Since NAD+ acts as substrate for specific NAD+-consuming enzymes, NAM is also generated as by-product by these reactions and recycled in the salvage pathway. Specifically, the cyclic ADP-ribose synthases (cADPRSs) hydrolyze NAD+ to NAM. Poly (ADP-ribose) polymerases (PARPs), use NAD+ as a co-substrate to PARylate target proteins (Subs), generating NAM. The deacetylation of specific substrates (Subs) by Sirtuins (SIRTs), which are NAD+ dependent enzymes, generates NAM. NAM is removed from recycling by degradation that thus indirectly affects NAD+ levels. NAM is methylated to 1-methyl-NAM (MNA) by NAM-*N*-methyltransferase (NNMT) and then oxidized to l-methyl-2-pyridone-5-carboxamide (2-Pyr) and l-methyl-4-pyridone-5-carboxamide (4-Pyr).

Nicotinamide (NAM) is an amide form of vitamin B3, a semi-essential vitamin. Many foods, including meat, fish, eggs, legumes, mushrooms, nuts, and grains contain NAM. However, NAM is also de novo synthetized (Figure 1) by tryptophan metabolism [1]. After ingestion, NAM is readily absorbed from the gastro-intestinal tract and widely distributed in the body tissues [2]. NAM is methylated in the liver to 1-methyl-NAM (MNA) by NAM-*N*-methyltransferase, and then oxidized to l-methyl-2-pyridone-5-carboxamide (2-Pyr) and l-methyl-4-pyridone-5-carboxamide (4-Pyr) (Figure 1). The major urinary metabolites are MNA and 2-Py, accounting for 20%–35% and 45%–60%, respectively [2].

Vitamin B3 deficiency affects tissues that need high cellular energy, such as brain, gut and skin causing pellagra. The main features of pellagra (photosensitive dermatitis, diarrhea, and dementia) are prevented by daily NAM intake of 15–20 mg from dietary sources [1,3,4]. NAM is the precursor of nicotinamide adenine dinucleotide (NAD+) (Figure 1), an essential co-enzyme of redox reactions for adenosine triphosphate (ATP) production and for several other metabolic processes [2,3,4]. Beyond its role as co-enzyme, NAD+ may act as substrate for specific NAD+-consuming enzymes and influences cellular responses to genomic damage through different mechanisms [2,4,5].

A two-step salvage pathway (Figure 1), which converts NAM to NAD+, represents the major route of NAD+ biosynthesis in mammals. NAM phosphoribosyltransferase (NAMPT) is the rate-limiting enzyme that catalyzes the first step in the biosynthesis of NAM mononucleotide (NMN) from NAM and ATP. Subsequently, the NMN adenylyltransferases (NMNATs) utilize ATP for the generation of NAD+. This coenzyme may be directly converted in NADP+ by NAD kinase [3,4]. NAD+ can be also synthetized by other precursors, such as nicotinic acid (NA) and NAM riboside (NR) (Figure 1).

NAD+ status is critical in preserving genomic stability. Tissues with high cellular turnover, such as skin, require higher doses of NAD+ to counteract genomic insults [6]. Low NAD+ level increases sun sensitivity in the skin. Indeed, it alters p53 expression, induces genomic instability, and reduces survival following exposure to solar-simulated (ss) UV radiation [3,6]. In animal models, NAD+ deficiency increases skin sensitivity to UV radiation, impairs DNA damage response, increases genomic instability, and increases cancer incidence [1,3,4]. Notably, elderly people more frequently display NAD+ depletion, and they are more prone to skin cancer [4]. Furthermore, NAD+ levels in skin are negatively associated with malignant phenotypes of tumors [6]. Thus, NAM may be involved in preventing skin damage, and consequently cancer, by influencing several processes such as reduction of DNA damage and optimization of DNA damage response.

Although NAM has been used for the treatment of a wide range of dermatological diseases, including autoimmune blistering disorders, acne, rosacea, and atopic dermatitis [3,7], the present review is focused on its role in genomic stability and skin cancer chemoprevention.

## 2. The Influence of NAD+ Status on Genomic Stability and Cell Senescence

Endogenous (e.g., oxidative metabolism, spontaneous hydrolytic reaction, DNA replication errors) and exogenous (e.g., UV and infrared radiations, chemicals) insults induce DNA damage, such as strand breaks, depurination, depyrimidination, crosslinks, and modified bases [8]. Genome stability is maintained by DNA repair systems (e.g., base excision repair (BER), nucleotide excision repair (NER), mismatch repair systems [9]. Following DNA damage, the cells undergo transient arrest to repair modifications through DNA repair systems, and then re-enter the cell-cycle [10]. The efficiency of mechanisms involved in genomic stability may depend on energy status. Indeed, several proteins, which contribute to repair DNA damage, such as DNA ligases (e.g., DNA ligase I, III), chromatin remodelers (e.g., CSB, Swi2/Snf2) and histone-modifying enzymes (e.g., ATM kinase, DNA-PK) are ATP-dependent enzymes [11]. Incomplete DNA repair leads to an accumulation of DNA damage that, in turn, leads to persistent DNA damage response, telomere attrition, and genomic instability. Persistent DNA damage has immediate effects, driving cells towards apoptosis or senescence, as well as long-term consequences resulting in aging or cancer [8,10]. Apoptosis and senescence are two tumor-suppression mechanisms that eliminate the cells at risk for oncogenic transformation. Apoptotic cells are quickly eliminated. Conversely, senescent cells remain viable in the tissue although they may undergo several changes, including growth arrest, modification of gene expression and morphology, and acquisition of a senescence-associated secretory phenotype (SASP) that is characterized by secretion of pro-inflammatory cytokines, growth factors, and proteases [12]. SASP allows the removal of damaged cells by recruiting immune cells, and promotes tissue renewal by mobilization of nearby progenitor cells. Thus, cell senescence protects the organism from developing cancer.

### 2.1. NAD+ Influences Energy Production by Acting as Co-Enzyme

NAD+ is a co-enzyme in several redox reactions which release energy from oxidized nutrients (e.g., glucose, fatty acids). The energy is transferred from NAD+ to NADH by reduction, as part of glycolysis, fatty acid β-oxidation, and the tricarboxylic acid cycle processes. NADH is oxidized to NAD+ by anaerobic glycolysis and mitochondrial oxidative phosphorylation (OXPHOS) that generates ATP. NAD+ is also a substrate for the biosynthesis of NADP+ by NAD kinase (NADK)-mediated phosphorylation. NADP+ has an analogous role for electron transfer in biosynthetic reactions such as fatty acid and cholesterol synthesis. Moreover, NADPH provides reducing equivalents to regenerate the cellular detoxifying and antioxidative defense systems. NADPH is also a substrate of NADPH oxidases (NOXs) that generate reactive oxygen species (ROS). Thus, the NAD(P)+/NAD(P)H ratio controls cellular redox status [2,5,13].

NAD+ level is critical in preserving genomic integrity of the cells, since DNA repair mechanisms are highly energy-dependent [11]. In human HaCaT keratinocytes, NAD+ depletion increased spontaneous DNA damage through up-regulation of NOX activity and subsequent increase in ROS production [14]. ROS increase induces DNA lesions, such as 8-oxo-2’deoxyguanosine (8-oxo-dG), that may result in GC to TA transition. This modified base is removed by the 8-oxoguanine-DNA glycosylase 1 (OGG1), an enzyme of the BER system [9]. NAD+ replenishment by NAM treatment completely reversed ROS accumulation and DNA damage in skin cells [14].

Low NAD+/NADH ratios promote cellular senescence, at least in part, by limiting glycolysis and ATP production. Indeed, senescent cells display decreased cytosolic NAD+/NADH ratio and increased AMP/ATP and ADP/ATP ratios. Lower energetic status activates the AMP-activated protein kinase (AMPK) that phosphorylates and activates p53, and, in turn, promotes senescence but suppresses the IL1R signaling arm of the SASP. AMPK also inactivates HuR, which increases the stability of transcripts of p21 and p16INK4a—two cyclin-dependent kinase inhibitors that mediate the senescence proliferative arrest through the activation of pRB tumor suppressor [13]. Furthermore, NAMPT inhibition induces senescence without the IL1R arm of the SASP [15]. Conversely, NAMPT overexpression suppresses cell senescence [16].

### 2.2. NAD+ Influences Cell Signaling by Acting as Substrate of NAD+-Consuming Enzymes

NAD+ may regulate genome stability and senescence acting as a substrate for the poly-ADP-ribose polymerase (PARP) enzymes, NAD+-dependent deacetylases of the Sirtuin family (SIRTs), and cyclic ADP-ribose synthases (cADPRSs), which can function as NAD-glycohydrolases (Figure 1). PARPs and SIRTs are enzymes that accomplish two key posttranslational modifications, i.e., poly-ADP-ribosylation (PARylation) and acetylation, respectively. Therefore, all these enzymes share NAD+ as common substrate and compete for its consumption. Moreover, these reactions release NAM that can be an endogenous inhibitor of both PARPs and SIRTs [2,4,17].

PARP-1 is the most common PARP, and is ubiquitously expressed. PARP-1 is as sensor of DNA breakage and has several roles in DNA damage responses, including DNA repair, maintenance of genomic stability, transcriptional regulation, signaling pathways involving apoptosis, telomere functions, and other multiple cellular functions [18,19]. PARP-1 is involved in maintaining a compact status and preventing accidental transcription [20]. Activated PARP-1 may favor DNA damage repair by either physical association or by PARylation of specific proteins, including single-strand break-repair proteins, BER enzymes, and itself. PARP-1 is activated by binding to DNA strand breaks. The PARylation of proteins proximal to breaks modifies their structure favoring the opening of the condensed chromatin and the recruitment of DNA repair complexes [19]. Furthermore, PARP-1 plays a key role in DNA repair through its association with NER and BER enzymes [17,19,21]. The intensity of DNA damage determines PARP-mediated cell response. Following mild or moderate DNA damage, activation of PARP-1 leads to DNA repair and restoration of normal cellular function [4]. When the damage is irreparable, PARP-1 facilitates apoptosis, preventing ATP depletion and DNA repair through PARP-1 caspase-mediated cleavage. However, persistent DNA damage leads to over-activation of PARP-1 and increased NAD+ catabolism causing suppression of NAD+-dependent ATP generation, energy crisis, and necrotic cell death [22].

SIRTs are NAD+-dependent deacetylase enzymes that remove acetyl, succinyl, and other similar groups from proteins. SIRTs controls several cellular processes, including transcription through histone epigenomic modulations, mitochondrial biogenesis, inflammation, cellular resistance to genotoxic insults, and metabolic dysfunctions [23,24]. SIRT1 plays a critical role in skin homeostasis, aging, and cancer [23]. The increase of the NAD+/NADH ratio enhances SIRT1 activity resulting in deacetylation of its targets, including the tumor suppressor protein p53 and the FOXO forkhead family of transcription factors. Conversely, low NAD+ levels decrease SIRT activity [23,25]. Cell survival following DNA damage may depend on SIRT1 activation intensity. p53 drives transcription of several genes triggering cell-cycle arrest, apoptosis, or DNA repair and its activity increases following DNA damage [23]. Moderate SIRT1 activation prevents apoptosis through p53 deacetylation that leads to its inhibition. However, continuous SIRT1 activation may induce cell death by accelerated NAD+ depletion [26].

Following genotoxic stress, PARP-1 and SIRT1 antagonistically interplay regulating energetic homeostasis, genomic integrity and inflammation. Indeed, they share NAD+ and several other substrates, including DNA repair enzymes and NF-kB. Persistent PARP-1 activation can either reduce NAD+ levels and raise NAM level, thus inhibiting SIRT1 activity. Similarly, the activation of SIRT1 reduces PARP activity. Moreover, PARP-1 inhibition prevents NAD+ depletion, restores the ATP levels, and activates SIRT1. Following oxidative stress, PARP-1 directly binds OGG1 that, in turn, stimulates PARP-1 activity. Moreover, activation of PARP-1 negatively regulates OGG1 activity [27]. Conversely, SIRT1 binds OGG1 and induces its deacetylation probably deactivating OGG1 when oxidative DNA repair is complete [28]. Furthermore, PARP-1 activates NF-kB that induces transcription increase of SASP components. On the contrary, SIRT1 suppresses the SASP through the deacetylation of lys310 residue of RelA/p65 subunit of NF-κB, which is critical for NF-κB transcriptional activity, and of histones in IL-6 and IL-8 promoter regions [17,21]. Notably, SIRT1 also interacts with PARP-1 by reducing its acetylation that is required for full NF-kB-dependent transcriptional activity. Conversely, SIRT1 can be PARylated by PARPs [17,21].

Overall, NAD+ is required for several cellular processes including ATP production, DNA repair and inflammation that are mediated by PARP-1 and SIRT1 activity. Critical depletion of NAD+ can result in cell death [2,4,26]. Moreover, PARP-1 and SIRT1 may have beneficial or detrimental effects on cell survival depending on the intensity of their activation.

Thus, the maintenance of adequate intracellular NAD+ levels and properly SIRT1 and PARP-1 activity is important in preventing DNA damage, genomic instability, and chronic inflammation that seems to be the major contributor to the cancer incidence increase in elderly [17,29,30].

## 3. Genomic Instability—Hallmark of Aging and Cancer

Genomic instability refers to an increased tendency of DNA alterations (e.g., point mutation, deletion and insertion, and chromosomal rearrangements) that irreversibly change genomic content and are one of hallmarks of both aging and cancer [8,10].

DNA damage is a driver of genome instability. An age-related increase of oxidative stress and/or decline of DNA repair activity could lead to an accumulation of DNA damage. Incorrect DNA repair or incomplete restoration of chromatin after damage repair, induce mutations or epimutations that accumulate with age [10].

Senescence protects against cancer but causes depletion of stem cell reservoirs and, in the long term, leads to tissue homeostasis decline by SASP that alters the functions of neighboring cells. Age-related deterioration of biological functions, which results in decline of DNA repair capacity and genomic instability, decline of immune system function and chronic inflammation, may contribute to a reduction of the efficacy of senescence response in counteracting tumorigenesis and, in turn, may lead to accumulation of senescent cells in tissues [10,31]. Studies in mouse models show a causative role of cellular senescence in driving aging in vivo [32,33].

Furthermore, the tumor-suppressor function of senescence response may turn into a tumor-promoting mechanism as a consequence of aging. Aged tissues are characterized by the accumulation of both DNA damage and senescent cells, as well as by the presence of SASP-mediated chronic inflammatory status [10]. Cellular senescence is a dynamic and progressive process, and therefore the properties of senescent cells continuously evolve and diversify. The age-related decline of cellular functions can delay the progression of the senescent program and the unstable state of the genome may allow to accumulate additional mutations [34]. Beyond promoting tissue repair and activating immune surveillance, SASP may also promote chronic inflammation, epithelial to mesenchymal transition, tumor immune evasion, angiogenesis or stem cell-like phenotype in malignant cells [30]. Age-related oncogenic stimuli, such as ROS increase and chronic inflammation, might induce damaged senescent cells to re-enter the cell-cycle and progress towards malignant transformation [34]. Notably, senescent cells accumulate in premalignant lesions but are not detectable after progression to malignancy [30,31,35]. Thus, malignant cells could derive from damaged cells escaping the senescence program, from cells damaged by neighbouring senescent cells through exacerbated pro-inflammatory secretion, or from senescent cells themselves. Indeed, senescent cells could be considered pre-malignant cells. Recent findings provide evidence regarding this issue: they indicate that the growth arrest of senescent cells can be bypassed upon the inactivation of key tumor suppressor genes, such as p16INK4a [30,34,36]. Therefore, some mutant genotypes, such as inactivation of tumor suppressor genes or oncogene overexpression, selectively favor the growth of cell subclones in a microenvironment suitable for tumor development created by SASP-mediated chronic inflammation.

Oxidative stress has a key role in epithelial senescence as it is responsible, at least in part, of the DNA damage and genomic instability that induces both p16INK4a/pRb-mediated cell-cycle arrest and SASP increase. Furthermore, oxidative stress is maintained by positive feedback loop involving SASP itself, p38 MAPK pathway and mitochondrial metabolism. Genome instability due to continuous oxidative damage enables cells to acquire malignant capabilities. Thus, epithelial cell senescence may be a tumor promoting mechanism that generates a reservoir of tumor initiating cells and produces an optimal niche for tumor progression [30,37]. Accordingly, the most frequent human tumors arise from epithelial cells, and their incidence increases with increasing age [38].

Thus, aging is a major risk factor of skin tumor development.

## 4. NAD+ Status and Skin Aging

**Figure 2:**
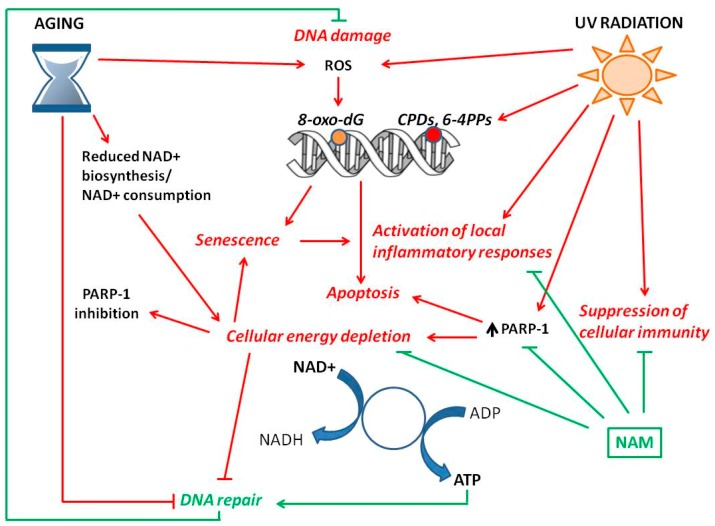
Nicotinamide (NAM) and genomic stability. The increase of reactive oxygen species (ROS) and the decrease of DNA repair capacity characterize skin aging and result in oxidative DNA damage with high levels of 8-oxo-dG lesions. Reduced NAD+ biosynthesis and/or NAD+ consumption induce cellular energy depletion that lead to decreased DNA repair activity and PARP-1 inhibition. Both NAD+ depletion and DNA damage can induce cell senescence or apoptosis. Senescent cell accumulation in the tissue is associated with chronic inflammation. UV radiation causes in the skin direct and indirect damages to DNA including cyclobutane pyrimidine dimers (CPDs) and pyrimidine (6–4) pyrimidine photoproducts (6–4PPs) and 8-oxo-dG lesions. UV rays also induce cellular energy depletion by increasing PARP-1 activity, activation of inflammatory responses and suppression of cellular immunity. NAM is a precursor of NAD+. Replenishment of the NAD+ pool increases adenosine triphosphate (ATP) production, counteracts the inflammatory responses and the suppression of immunity. Furthermore, the ATP increase enhances DNA repair mechanisms maintaining genomic stability. Therefore, NAM protects the skin from DNA damage and may have beneficial effects against skin aging and tumor development.

NAD+ depletion has been associated with hallmarks of aging (Figure 2). The maintenance of adequate NAD+ levels may be beneficial in counteracting at least some of the age-related cellular degenerative processes [2,4,5].

### 4.1. NAD+ and Chronological Skin Aging

Chronological or intrinsic skin aging results from the passage of time and is mainly influenced by genetic factors and metabolic processes. Indeed, specific skin aging signs, such as mitochondrial dysfunction, genomic instability, cellular senescence, and the breakdown of the extracellular matrix (ECM) are a consequence of dysfunctional oxidative processes [39].

Chronologically aged skin is characterized by epidermal atrophy that mainly affects the stratum spinosum and is due to lower epidermal turnover rates [40]. The loss of stem cell function can result, in part, from genomic instability and senescence. As the levels of NAD+ [41] and BER activity [42] decrease with epidermal aging, the DNA integrity can be compromised. Accordingly, an accumulation of 8-oxo-dG has been observed in aged primary keratinocytes. Notably, 8-oxo-dG accumulation displays a positive linear relationship with the expression of the skin aging biomarker p16INK4a and stem cell depletion [42]. Furthermore, NAD+ depletion also increases NOX-mediated ROS production that can be counteracted by NAM administration [14].

The inhibition of NAM conversion to NAD+ in primary human keratinocytes reduces the rates of glycolysis and OXPHOS, and induces cell senescence indicating an instrumental role of NAD+ status in the balance of epidermal proliferation/differentiation [43]. Indeed, NAM administration prevents the differentiation of keratinocytes and enhances their clonogenicity and proliferation [43].

Alteration to the oxidative and energy metabolism has a causative role also in NER disorders. Xeroderma pigmentosum (XP) is caused by deficiency of specific NER enzymes (XPA-XPG) and is characterized by premature aging and high skin cancer incidence. The XPC enzyme has a protective role against oxidative DNA damage [44,45]. XPC silencing in normal human keratinocytes induces the activation of NOX that lead to redox unbalance and mitochondrial dysfunction [45,46]. NOX1 activation in XPC-deficient cells triggers premature skin aging and neoplastic transformation of keratinocytes. Inhibition of NOX1 activity rescues premature skin aging signs in young *Xpc-/-* mice [45].

Aged dermal fibroblasts display reduced NADPH/NADP+ redox ratio and collagen secretion compared to those from young donors. NAM administration induces a significant increase of collagen biosynthesis with significant positive effects in counteracting skin aging and photodamage [47].

An age-related NAD+ decrease could be due to decreased biosynthesis and/or increased NAD+ consumption. The biosynthetic enzyme NAMPT expression decreases in several tissues during aging, thus leading to NAD+ deficiency, reduced Sirt1 activity and, in turn, to cell senescence [5]. NMN and NAM administration induces restoration of NAD+ levels and counteracts senescent signs.

Concomitant with NAD+ depletion, PARP1 function declines with aging in human and mice [4,41]. Decreased NAD+ levels lead NAD+-binding protein DBC1 to form a complex with PARP1, inhibiting its catalytic activity [48]. NMN and NAM treatment breaks DBC1-PARP1 complexes, restores PARP1 activity, and attenuates age-related DNA damage in aged mice [49,50]. Moreover, SIRT1 activity is negatively associated with skin age of men but not of women [41]. However, significant decreases in NAD+ levels and SIRT1 activity have been observed in aged female rats [51]. SIRTs are involved in the cellular response to prevent oxidative stress in the skin [23]. Moreover, the loss of SIRT1 disrupts skin barrier integrity in mice [52]. Although the role of SIRT1 in cancer development is still under debate [23], SIRT1 levels are significantly reduced in human skin tumors, suggesting that SIRT1 may act as a tumor suppressor through its role in DNA repair [53].

### 4.2. NAD+ and Photo-Aging

As skin protects the body from environmental insults (e.g., UV radiation, pollutants, xenobiotics, atmospheric oxygen) that induce increases in ROS production, extrinsic aging has a primary role -as well as intrinsic aging- in damaging the tissues. Thus, aged skin can reflect different stages of extrinsic aging, superimposed on intrinsic changes [39]. Acute or repeated sun exposure induces short-term skin damages such as sunburn, characterized by skin erythema. Long-term sunlight exposure to sub-erythemal doses is associated with photo-aging or UV-induced skin cancers [54].

Although keratinocytes are continuously hit by radiations, in aged skin UV-exposure is elevated due to compromised stratum corneum. The concomitant age-related NAD+ deficiency and increased requirements of NAD+ for DNA damage response may contribute to energy stress that generates additional ROS and elevated genomic damage [3].

UV radiation causes several kinds of damage in skin cells (Figure 2): (i) DNA damage; (ii) reduction of DNA repair capacity; (iii) activation of local inflammatory responses; (iv) suppression of cellular immunity [55].

Both types of UV radiation induce DNA damage. UV-B rays (290–320 nm), which account for about 0.5% of solar spectral irradiance reaching the earth, are highly energetic and damage epidermal cells. UV-B radiation induces direct DNA damage by forming cyclobutane pyrimidine dimers (CPDs) and pyrimidine (6–4) pyrimidine photoproducts (6–4PPs) that are removed primarily by NER system [9,55]. These DNA lesions, if not effectively repaired, may result in genetic mutations. CPDs give rise to C→T and CC→TT transitional mutations that may alter the function of the p53 gene, which is important as tumor suppressor in skin carcinogenesis [8]. An increase of these transitional mutations has been found in skin tumors of both animal models and human patients [56]. Conversely, UV-A rays (320–400 nm) correspond to 95% of UV radiation at ground level. They are less energetic but have higher penetration properties so that they mainly induce direct dermal alterations through the ROS generation and subsequent oxidative DNA damage. Notably, Nox1 is the major source of UVA-induced ROS in human keratinocytes [9,55].

UV-induced ATP depletion may hamper DNA repair capacity. Studies performed in mouse models and cultured human keratinocytes demonstrate that UV-B deplete NAD+ making cells more sensitive to DNA damage and prone to tumorigenesis [4]. SIRTs and PARPs play an important role in resistance to photo-damage [57]. Low-dose, sub-erythemal UV-B activate PARP-1 activity, at least partially, via UV-induced oxidative DNA damage [22]. Excessive activation of PARP-1 blocks glycolysis, reduces intracellular energy in keratinocytes, and leads to cell death. Increased activity of PARP-1 depletes the NAD+ pool, leading to reduced activity of SIRTs [4,5]. Notably, SIRT1 confers protection against UV-B radiation- and ROS-induced skin cell damage [57]. Adequate NAD+ levels are critical for proper DNA repair upon UV exposure. NAD+ deficiency increases skin sensitivity to photo-damage, whereas NAM prevents UV-induced depletion of cellular energy by replenishing the NAD+ pool [3]. NAM is also a PARP inhibitor. Thus, NAM increases intracellular ATP levels by inhibiting the PARP-1 mediated blockade of the glycolytic pathway that, in turn, replenishes NAD+ and ATP reservoirs [58]. Furthermore, NAM enhances repair of UV-induced DNA damage. HaCat cells treated with NAM and then exposed to UV display initial levels of CPD and 8-oxo-dG photolesions similar to untreated cells, indicating that NAM does not reduce or prevent DNA damage induction [59]. Moreover, NAM did not restore the ROS steady-state levels after UV-treatment, and hence does not reduce 8-oxo-dG via direct antioxidant effects [58]. However, an increased amount and rate of DNA excision repair has been found in UV-irradiated NAM-treated cells [59]. NAM enhanced DNA repair also in old mice [4]. Thus, the increasing of ATP production in UV-irradiated cells enhances DNA repair capacity, and may reduce mutation frequency.

UV radiation activate local inflammatory responses. UV-B rays lead to the production of pro-inflammatory cytokines which induce inflammation and cell death. The cytokines produced by UV-B-irradiated keratinocytes include IL- 1, IL- 6, IL- 8, IFN- γ, granulocyte colony- stimulating factor (C- GSF), macrophage inflammatory protein (MIP- β), and TNF- α. [1,60]. IL-1α and IL-6 induce the secretion of MMP-1, which hydrolyzes type I collagen thus playing a crucial role in the disorganization and progressive degeneration of dermal ECM, by a paracrine mechanism [61,62]. UV-A radiation stimulates fibroblasts to produce and release MMP-1 and elastase that lead to drastic alterations of dermal structure and “solar elastosis” [63,64]. Notably, photo-aged fibroblasts contribute to skin hyperpigmentation, which is associated with photo-aging, by the paracrine secretion of melanogenic cytokines [65]. NAM reduces UV-induced inflammation in cell cultures and mouse models. Indeed, UV-irradiated NAM-treated HaCat cells showed significant downregulation of IL-6, IL-10, MCP-1 and TNF-α mRNA expression, indicating effectiveness in suppressing local inflammatory responses. Moreover, PARP-1 enhances the expression of various pro-inflammatory mediators in mice. NAM exerts anti-inflammatory effects via the PARP pathway [66]. However, clinical studies report that oral or topical NAM administration did not reduce the erythema caused by UV radiation [67,68].

UV radiation at sub-erythemal doses suppresses cutaneous cellular immunity. Both UV-B and UV-A rays are immune suppressive [1,69]. The immune system is a key defense mechanism in destroying mutant keratinocytes and preventing tumorigenesis. UV-induced DNA damage, mainly CPDs, triggers both the suppression of immune responses and initiation of carcinogenesis [67,70]. The immune response is suppressed by several mechanisms, including decreased tumor antigen-presenting cell function and the increased production of immunomodulatory cytokines [71].

NAM prevents immunosuppressive effects of UV radiation in mouse models, both when administered topically or orally [72,73]. Moreover, topical NAM significantly protected against immunosuppression caused by UV-B, longwave UV-A, and single and repeated ssUV exposures in humans [74,75]. Double-blind, randomized, placebo-controlled crossover trials showed that oral NAM administration (from 500 mg once daily to 500 mg thrice daily for 7 days) reduces the level of immunosuppression induced by UV radiation [67]. Notably, NAM exerts an immune-protective role against both UV-A and UV-B. Moreover, NAM did not enhance baseline immunity at unirradiated sites, indicating that it normalizes immune responsiveness only in UV-irradiated areas [67]. Moreover, oral NAM (500 mg twice daily) significantly reduced trans-epidermal water loss in 292 participants with multiple previous skin cancers [76].

### 4.3. NAM and Prevention of Aging Signs

Following the significant and replicable benefits of NAD precursors in aged animal models [5], several clinical trials in healthy elderly humans have been performed.

In a double-blind, randomized, controlled trial 5% NAM cream was applied twice daily for 12 weeks to half of the face, and its vehicle control to the other half of the face, of 50 Caucasian women. NAM treatment significantly improved skin appearance by reducing signs of aging, including reductions in fine lines and wrinkles, hyperpigmented spots, red blotchiness, and skin sallowness, and it improved skin elasticity [77]. Another double-blind, split-face, randomized controlled study was performed in 30 healthy Japanese females. Therein, 4% NAM was applied for eight weeks in areas around the eyes. NAM treatment significantly improved wrinkle grades and skin roughness [78].

NAM inhibits melanosome transfer from melanocytes to keratinocytes, thus promoting skin lightening in vivo [79]. A double-blind, full-face, randomised controlled trial was conducted in 202 women aged 40–60 years using topical formulation containing 2% *N*-acetyl glucosamine and 4% NAM. The treatment significantly reduced the appearance of irregular pigmentation including hyper-melaninization [80]. A double-blind, split-face, randomized controlled study was performed in 40 women aged 40–70 years to evaluate the effects of topical application of a gold Silk Sericin/NAM/Signaline complex on biophysical parameters related to skin aging. Significant improvements in stratum corneum hydration, barrier function, and elasticity were observed [81].

Taken together, these studies indicate that NAM is able to recover several skin chronological and photo-aging signs in cell cultures, mouse models, and clinical studies on humans [3,4,5].

## 5. NAD+ Status and Skin Cancer

NAD+ depletion with age and/or following UV radiation may play a major role in skin cancer initiation by impairing processes involved in genomic stability. Thus, the maintenance of adequate intracellular NAD+ levels is important for skin cancer prevention [4].

### 5.1. Cutaneous Squamous-Cell Carcinoma

Cutaneous squamous-cell carcinoma (cSCC) is one of the most common cancers in Caucasian populations, representing 20% of all cutaneous malignancies. It usually arises from the in situ lesion actinic keratosis (AK), or it may grow de novo in photo-exposed areas. The main known risk factors are advanced age, cumulative sun exposure, fair skin, prolonged immunosuppression, human papillomavirus infection, male gender, and previous diagnosis of cutaneous tumors [82,83]. Outdoor workers and people with weakened immune systems by therapies or chronic infectious diseases have a higher risk of developing cSCCs [82].

AK is a limited area of irregular epidermal growth, caused by excessive solar exposure, visible as a thickened, cornified, scaly lesion characterized by keratinocyte atypia. The degree of intraepidermal involvement by this atypia is graded as mild (AKI), moderate (AKII), or severe (AKIII). AKIII is considered as an in situ cSCC (iSCC) [84]. Indeed, AK and cSCC are stages of a continuous multistep process that derives from p53 gene mutation and proliferation of atypical keratinocytes. Cumulative sunlight exposure, impaired DNA repair, immune response dysregulation, and changes in the surrounding stroma favor this transition and the disease progression. Clinical studies have estimated that 65% of cSCCs emerge from the site of an AK lesion [85]. Moreover, up to 16% of AKs progress to invasive cSCCs, with extrapolation studies which estimate the risk of progression at 10%. This progression takes approximately two years [83]. Thus, if left untreated, AK may often progress to cSCC, thus causing increased morbidity [85]. The exact incidence rate of AK and cSCC is unknown because in most countries these lesions are not reported in cancer registries. The highest prevalence of AK is reported from Australia (up to 60%), an area with intense UV irradiation home to a large fair-skinned population. In Europe, a prevalence of 15% in men and 6% in women has been reported. In the same study, 34% of males and 18% of females aged 70 years or older had AKs [38]. In Europe, cSCC showed a marked geographic variation with the highest incidence rates in South Wales (31.7 per 100,000 person-years) and Switzerland (28.9 per 100,000 person-years) [38]. Invasive cSCC has the potential to recur (8%) and metastasize (5%) [86]. AKs can occur as single lesion or arise from the field cancerization (FC), a chronically photo-injured skin area in which clinical and subclinical lesions coexist. The presence of multiple AKs is a marker of increased risk of developing invasive cSCC, and early treatment of AKs reduces the probability of progression [38,83,84].

As the individual risk of progression from AK to invasive cSCC cannot be accurately predicted, it is preferable to treat the entire FC. Indeed, the clinical grading of AKs has scarce prognostic value, although it may be useful in guiding treatment choices [85]. Different approaches are available to treat AKs. Ablative procedures such as cryosurgery, curettage, laser ablation, and surgical excision are generally used to treat single lesions; many topical treatments (e.g., photodynamic therapy, 5-fluorouracil (FU), ingenol mebutate, diclofenac 3% gel, and imiquimod) may be employed to treat both individual lesions and the FC. At 12-month follow-up, the reported sustained clearance rate of initially cleared individual AKs is 28% for cryosurgery, 54% for 5-FU, 59% for PTD, 73% for imiquimod, 81% for diclofenac 3% gel, 87% on face and scalp or trunk and extremities for ingenol mebutate. However, these treatments are poorly effective on AKIII lesions and FC [84].

Chemoprevention represents the use of pharmacological interventions to potentially interrupt the development of new malignancies. Indeed, chemopreventive agents might reduce the incidence of AKs in the FC by inhibiting the early effects of UV radiation. They are becoming a viable strategy to prevent or reduce AK relapse and cSCC occurrence [85].

### 5.2. NAM and Chemoprevention

Oral NAM at doses of 500 mg twice daily and 500 mg once daily was evaluated in two double-blind, randomized, placebo-controlled phase two trials in Australians with sun-damaged skin with an average of more than 30 AK at baseline. Within four months, significant relative reductions in AK of 35% and 29% with twice daily and once daily NAM dosing have been observed, respectively [87]. A phase three randomized controlled trial was carried out on 386 Australians that had had at least two non-melanoma skin cancers (NMSC) in the previous five years. The study showed that oral NAM treatment (500 mg twice daily for 12 months) is safe and effective in reducing the rates of new NMSCs and AKs. Patients receiving NAM showed a 23% relative rate reduction in new NMSCs (basal cell carcinoma (BCC) and SCC) compared to placebo (*p* = 0.02); the unadjusted relative rate reduction was 27% (*p* = 0.02). There were similar magnitudes of reduction for both BCC (relative rate reduction 20%, *p* = 0.12) and SCC (relative rate reduction 30%, *p* = 0.05). No difference in NAM effectiveness in reducing cSCC in situ versus well-differentiated and versus moderately-differentiated was observed [88]. Notably, a significant decrease in the number of macrophages was observed in those lesions arising in NAM treated-patients compared to placebo [89]. AK counts followed the same trend. Significant relative reductions in AK counts were observed throughout the intervention period at three, six, nine, and 12 months. NAM effectiveness in reducing AK counts appeared to plateau after the nine-month visit (relative rate reduction 20%, *p* < 0.001). However, there is no evidence of benefit after NAM is discontinued [88]. Any significant effects of oral NAM on cognitive function nor on quality of life have been found [90].

Two small phase two randomized controlled trials indicated that NAM may also have chemoprotective effects in immune-suppressed transplant recipients. Thirty renal transplant recipients received placebo or nicotinamide 250 mg thrice daily for six months and found reductions in AK without detectable effects on the blood levels of immune-suppressed patients [91]. Twenty-two renal transplant recipients were treated with placebo or NAM 500 mg twice daily for six months and found non-significant trends to reduction in new skin cancers and AK over the six-month intervention period without significant increase in adverse effects nor significant change in blood or urine parameters or blood pressure [92].

Unlike NA, oral NAM does not cause vasodilatory side-effects, such as flushing, headache, hypotension, and itch, and has been well tolerated at high doses (1.5–3 g daily) for a range of clinical and research indications including inflammatory skin diseases [93], as a radio-sensitizer [94], and in diabetes prevention studies in children and adults [95]. NAM has an established safety profile even at high doses, although a reversible elevation of liver enzymes and nausea have been reported in some patients at much higher daily doses (8 g daily) [1]. The phase three clinical trial included patients ranging in age from 30 to 91 years (mean 66 years), many with multiple comorbidities and receiving numerous concurrent medications [88]. The 86 serious adverse event reports, were evenly distributed across the placebo and NAM arms, with no notable between-group differences in adverse events nor in blood pressure or bodyweight (measured quarterly for 12 months), full blood count, renal function, or hepatic function (measured at baseline and at 12 months) [88]. Systemic absorption of topically delivered NAM has been reported to be approximately 10% depending on the vehicle used [96]. NA and its derivatives applied topically may act also through the nicotinic acid receptor signaling inducing keratinocyte differentiation [3].

NAM inhibits phosphate co-transport in the gut and in the renal proximal tubule, and it has been used as a phosphate-lowering agent in hemodialysis patients, at doses of 1–2 g daily [97]. Four cases of thrombocytopenia (to </mm^3^) have been reported among 49 hemodialysis patients taking an average of 1.3 g NAM daily, although there was no overall change in mean platelet count from baseline in patients taking NAM [97]. All cases of thrombocytopenia resolved after the withdrawal of NAM. This study also found an increased incidence of nausea and diarrhea with NAM, not seen in the phase three clinical study [88], which excluded patients with renal failure. NAM is renally excreted and hence may present a different side-effect profile in patients with renal impairment. NAM is able to cross the human placenta, and fetal blood levels of NAM are greater than the corresponding maternal blood levels. However, there is no evidence of growth retardation, teratogenicity, and oncogenicity in humans [1]. NAM has few established drug interactions. There is a possible interaction with carbamazepine, thus NAM may be best avoided in patients taking this medication [1].

## 6. Conclusions

NAD+ levels are critical for genomic responses to genotoxic insults [2,4]. Maintaining adequate NAD+ level is therefore essential in skin cancer patients and individuals at risk of exposure to genotoxic agents. Intracellular NAD+ levels are regulated by many cellular processes, including glycolysis, OXPHOS, mitochondrial metabolism, transcription and signaling, and are significantly influenced by diet, exercise, and other health conditions. However, NAD+ depletion is associated with aging and age-related diseases, including cancer. Recently, beneficial effects of NAD+ precursors (e.g., NR, NMN, NAM) in delaying signs of age-associated diseases have been found [2,5,88].

NAM prevents ATP depletion and glycolytic blockade induced by UV radiation boosting cellular energy and enhances DNA repair activity in cultured keratinocytes and animal models [3,4]. Thus, NAM is involved in the maintenance of genomic stability and may have beneficial effects against aging-related skin changes and tumor development [1,4]. NAM reduces skin cancer incidence and prevents the immune-suppressive effects of UV radiation in mice when given topically or orally [72,73]. Furthermore, clinical studies have shown that topical use of NAM reduced skin aging signs [77,78,79,80]. Oral NAM administration reduces the level of immunosuppression induced by UV radiation without altering baseline immunity and it lowers the rate of NMSCs, including AKs.

Therefore, a NAM replenishment strategy may be a promising approach for the chemoprevention of NMSC.

## Abbreviations

NAM	nicotinamide
NAD+	nicotinamide adenine dinucleotide
UV	ultraviolet
ATP	Adenosine triphosphate
